# Preparation of Antioxidant Peptides from Hippocampus Abdominalis via High Hydrostatic Pressure-Assisted Enzymolysis: Evaluation of Their Protective Effects Against Male Reproductive Dysfunction in Mice

**DOI:** 10.3390/antiox15101338

**Published:** 2026-10-16

**Authors:** Xiaoxu Zhang, Tingyu Feng, Mengjia Zhu, Jiangbo Xiang, Guanghua Xia, Xiaoming Jiang, Xiang Gao

**Affiliations:** 1 College of Life Sciences, Qingdao University, Qingdao 266071, China; 2 Qingdao Institute of Marine Bioresources for Nutrition & Health Innovation, Qingdao 266109, China; 3 Quanzhou Institute of Marine Bioresources Industry, 3rd Floor, China Shishi International Food City, No. 2023 Haining Road, Baogai Town, Quanzhou 362700, China; 4 School of Food Science and Technology, Hainan University, Haikou 570228, China; 5 China Resources Wind Power (Shanwei) Co., Ltd., Shanwei 516600, China; 6 State Key Laboratory of Marine Food Processing & Safety Control, College of Food Science and Engineering, Ocean University of China, No. 1299, Sansha Road, Qingdao 266003, China

**Keywords:** *Hippocampus abdominalis*, antioxidant peptides, high hydrostatic pressure, enzymatic hydrolysis, reproductive impairment, in silico screening, mechanism

## Abstract

*Hippocampus abdominalis* (*H. abdominalis*) is an important marine teleost fish with diverse functional activities. Accordingly, the present research was designed to isolate and prepare innovative antioxidant peptides derived from *H. abdominalis* and to assess the protective and restorative impacts of its protein hydrolysates (HAPH) against reproductive injury caused by triptolide (TP) in male mice. Notably, pepsin was ultimately identified as the optimal protease among seven screened proteases. High hydrostatic pressure (HHP) pretreatment significantly increased peptide yield and antioxidant activity of pepsin hydrolysates. Subsequently, the optimal enzymatic hydrolysis conditions were established as E/S of 3% (*w*/*w*), hydrolysis time of 3 h, S/L of 1:25 (*w*/*v*), HHP pressure of 200 MPa, and 9 min of HHP time using single-factor experiments and response surface methodology. The peptide fraction with a molecular weight below 3 kDa (HAPH-1) exhibited stronger antioxidant activity and excellent stability against pH, temperature, and simulated gastrointestinal digestion. Furthermore, animal experiments revealed that HAPH-1 alleviated triptolide-induced impaired spermatogenesis and testicular tissue damage in mice, and the underlying mechanisms involve the regulation of the Keap1-Nrf2 pathway and the Bcl-2/Bax/caspase-3 apoptotic signaling pathway. Finally, through LC-MS/MS analysis, molecular docking, and in vitro activity validation, three novel peptides with high antioxidant activity (GPAPWGF, FPALLF, and LPLPLF) were successfully screened. Collectively, this study provides new insights into the high-value utilization of *H. abdominalis* and the development of novel antioxidant functional components.

## 1. Introduction

Infertility, defined as the failure to achieve a clinical pregnancy after 12 months or more of regular unprotected sexual intercourse, has emerged as a serious global health issue. Currently, approximately 8% to 12% of couples in their reproductive years experience infertility across the globe, and male-related infertility accounts for 30% to 50% of all such instances [1]. Male reproductive dysfunctions, including azoospermia and testicular dysfunction, constitute the primary etiological factors of male infertility. Changes in modern lifestyle, such as exposure to pesticides, heavy metals, and industrial chemicals, as well as obesity, smoking and alcohol abuse, malnutrition, and sedentary lifestyle, have contributed to the growing prevalence of male reproductive dysfunction [2]. Therefore, the implementation of proactive measures to protect male reproductive health has become an urgent public health challenge. Currently, assisted reproductive technology (ART) and pharmacotherapy are the primary approaches for improving male reproductive disorders. ART refers to a therapeutic approach designed to meet the reproductive needs of couples experiencing infertility, mainly including in vitro fertilization (IVF) and intracytoplasmic sperm injection (ICSI) [3]. Nevertheless, ART increases the risk of metabolic diseases in later life among offspring by inducing alterations in placental development [4]. In addition, pharmacological therapy is frequently accompanied by adverse effects. For instance, sildenafil, used for the treatment of erectile dysfunction, may induce ocular disorders [5]. Therefore, exploring natural products with both reproductive-improving potential and excellent safety has become a critical research focus.

Bioactive peptides (BPs), typically consisting of 2 to 20 amino acids [6], have attracted considerable attention in improving human health owing to their remarkable bioavailability, potent biological activity, and high stability under various conditions [7]. Accumulating evidence indicates that bioactive peptides exert ameliorative effects against hormonal imbalance, infertility, sexual dysfunction, and erectile dysfunction [8]. Among these, antioxidant peptides exert a protective effect on damaged cells by scavenging free radicals and enhancing the activity of endogenous antioxidant enzymes, thereby holding great potential for improving male reproductive function [9]. For instance, Valizadeh et al. [10] isolated antioxidant bioactive peptides from traditional fermented fish sauce that mitigated heat stress (HS)-induced spermatogenesis arrest, germ cell depletion, and cellular apoptosis in testicular tissue of male rats.

Seahorses represent a group of marine teleost fishes taxonomically classified under the genus *Hippocampus* of the family *Syngnathidae.* They mainly inhabit temperate and tropical coastal waters, distributed from approximately 50° N to 50° S latitude [11]. To date, 32 species of seahorses have been identified worldwide [12,13]. Seahorses, an integral part of traditional medicine across multiple regions worldwide, exhibit a range of bioactive properties and are employed to manage conditions including gastritis, infertility, erectile dysfunction, goiter, and asthma [11]. In traditional Chinese medicine (TCM), seahorses are considered to have therapeutic effects against various ailments, like tonifying the kidney and strengthening yang, regulating qi, and promoting blood circulation [14]. The *H. abdominalis*, also called the big-belly seahorse, is the largest species of seahorse. Owing to its rapid growth rate and high adaptability, the *H. abdominalis* has become one of the most important farmed seahorse species globally [15]. Currently, *H. abdominalis* has obtained food use approval from the Korea Ministry of Food and Drug Safety, and has also been included in the food and drug catalogs for approved use in countries such as the Philippines [16]. Serious evidence indicated that *H. abdominalis* might be a promising source for the preparation of antioxidant peptides. *H. abdominalis* contains approximately 70% protein on a dry weight basis and is rich in hydrophobic amino acids including Ala, Pro, Leu, Val [17]. These hydrophobic amino acids frequently appear in antioxidant peptides, facilitating the interaction between peptides and free radicals [18]. Kim et al. [19] have prepared antioxidant peptides from *H. abdominalis* using alcalase and identified a potent antioxidant peptide (Ala-Gly-Asp), which effectively reduced intracellular reactive oxygen species (ROS) levels and alleviated cell death in zebrafish embryos induced by 2,2-azobis hydrochloride (AAPH). In addition, a few studies have demonstrated that peptides derived from *H. abdominalis* can improve male reproductive function. Kim et al. [20] reported that the enzymatic hydrolysates of *H. abdominalis* by pepsin and alkaline protease attenuated H_2_O_2_-induced apoptosis in mouse Leydig (TM3) cells and markedly increased sperm count and motility and elevated serum testosterone levels in normal ICR mice. Another study proved that protein hydrolysate derived from *Hippocampus kuda* exerted therapeutic effects on reproductive disorders in male diabetic rats, as evidenced by elevated serum concentrations of testosterone, follicle-stimulating hormone, and luteinizing hormone, as well as enhanced sperm quantity and viability [11]. Currently, no study has systematically prepared antioxidant peptides from *H. abdominalis* and evaluated their improvement effects on male reproductive dysfunction.

Enzymatic hydrolysis is widely recognized as an effective and safe method for preparing antioxidant peptides [21]. The development of efficient pretreatment methods, such as thermal treatment, high hydrostatic pressure, ultrasound, and microwave treatment, has become a research hotspot, as they can facilitate enzymatic hydrolysis and promote the release of novel bioactive peptides [22]. Among these technologies, HHP-assisted enzymatic hydrolysis has emerged as a superior strategy. By unfolding protein structures and increasing the exposure of enzymatic cleavage sites, HHP-assisted enzymatic hydrolysis markedly enhanced proteolytic efficiency and functional properties of the resulting hydrolysates [23]. HHP-assisted enzymatic hydrolysis promoted the release of antioxidant peptides from cowhide gelatin [24]. Zhou et al. [25] reported that HHP-assisted enzymatic hydrolysis markedly increased peptide yield and antioxidant activity of peptides from buckwheat globulin. To date, no study has prepared bioactive peptides from *H. abdominalis* through HHP-assisted enzymatic hydrolysis process.

The objective of this study was to prepare and identify novel antioxidant peptides from *H. abdominalis* through HHP-assisted enzymatic hydrolysis technology. Furthermore, the ameliorative effects and mechanisms of the acquired peptides on reproductive dysfunction in male mice were investigated. The findings will provide a theoretical foundation for the high-value utilization of *H. abdominalis* and the development of functional products against male reproductive dysfunction.

## 2. Materials and Methods

### 2.1. Materials

*H. abdominalis* was farmed and provided by Dongying Broaden Sea Aqua Tech Co. Ltd., Dongying, China. Neutral protease (2 × 10^5^ U/g), alkaline protease (2 × 10^5^ U/g), papain (2 × 10^5^ U/g), animal protease (1 × 10^5^ U/g), trypsin (2.5 × 10^2^ USPu/mg), pepsin (3 × 10^3^ U/g, NF) and collagenase (1 × 10^5^ U/g) were acquired commercially from Pangbo Biological Engineering Co., Ltd., Nanning, China. Triptolide (TP) were obtained from Shanghai Yuanye Biotechnology Co., Ltd. (Shanghai, China). 17-Methyltestosterone (MT) was purchased from Chengdu Doster Biotechnology Co., Ltd. (Chengdu, China). Chemicals including trichloroacetic acid (TCA), ABTS, and DPPH were acquired from Macklin Biochemical Co., Ltd. (Shanghai, China).

### 2.2. Protease Screening and Pretreatment Selection

Seven enzymes were selected to hydrolyze *H. abdominalis*: Neutral protease (pH 7.0, 45 °C), alkaline protease (pH 10, 55 °C), papain (pH 6.5, 55 °C), animal protease (pH 7.0, 50 °C), trypsin (pH 8.0, 37 °C), pepsin (pH 2.0, 37 °C), and collagenase (pH 7.5, 37 °C). *H. abdominalis* was pulverized using a disintegrator and then blended with deionized water at a solid–liquid ratio of 1:15 (*w*/*v*). Seven different proteases were separately introduced at an enzyme-to-substrate mass ratio of 3% (*w*/*w*). Each hydrolysis reaction was carried out under individually optimized temperature and pH values for 3 h. All enzymatic hydrolysis reactions were terminated by incubating the mixed solutions at 100 °C for a duration of 10 min. After centrifugation at 8000 rpm for 15 min, the resulting supernatant was collected and used for the determination of peptide production yield and DPPH free radical scavenging capacity.

To investigate the effects of HHP pretreatment on the hydrolysis of *H. abdominalis*. *H. abdominalis* was suspended in distilled water at a ratio of 1:15 (*w*/*v*). The resulting solution was treated as follows: (1) HHP treatment at 300 MPa for 6 min, (2) a control group with no pretreatment, and pepsin was added at an E/S of 3% (*w*/*w*). Hydrolysis was then performed under the optimum reaction conditions tailored for pepsin: pH 2.0 and 37 °C. The reaction was allowed to proceed for 3 h, terminated by thermal inactivation at 100 °C over a 10 min period. Following centrifugation at 8000 rpm for 15 min, the upper supernatant fraction was harvested and employed for the determination of peptide yield and DPPH radical scavenging capacity. All experiments were carried out in three independent replicates.

### 2.3. Optimization of Hydrolytic Conditions for H. abdominalis

#### 2.3.1. Single-Factor Experiments

A precisely weighed 5 g sample of *H. abdominalis* was employed, and the preparation procedure was optimized using the variables listed below: the HHP time (2, 4, 6, 8 and 10 min), the HHP pressure (100, 200, 300, 400 and 500 MPa), the E/S (1%, 2%, 3%, 4% and 5%), the S/L (1:10, 1:15, 1:20, 1:25 and 1:30), the duration of hydrolysis (1, 2, 3, 4 and 5 h). All experiments were performed in three independent replicates.

#### 2.3.2. Plackett–Burman and Response Surface Design

To optimize the hydrolysis conditions for *H. abdominalis* hydrolysates, a three-factor, three-level response surface design was applied following the identification of critical variables using the Plackett–Burman method informed by single-factor experiments. The Plackett–Burman trial considered variations in the HHP time (6, 10 min), HHP pressure (100, 300 MPa), E/S (2%, 4%), S/L (1:20, 1:30), and hydrolysis time (2, 4 h). Subsequently, the response surface design was carried out with the following ranges: HHP time (6, 8, 10 min), E/S (2%, 3%, 4%), S/L (1:20, 1:25, 1:30). All experiments were performed in three independent replicates.

### 2.4. Membrane Ultrafiltration of H. abdominalis Hydrolysate

HAPH were prepared under the established optimal hydrolysis conditions. Subsequently, the resulting supernatant was isolated using an ultrafiltration setup equipped with a membrane possessing a 3 kDa molecular weight cutoff, employing a Pellicon XL system manufactured by Millipore (Burlington, MA, USA). Two distinct fractions were harvested, designated as HAPH-1 (molecular weight below 3 kDa) and HAPH-2 (molecular weight above 3 kDa), and their DPPH radical scavenging capacities were evaluated at a peptide concentration of 5 mg/mL.

### 2.5. Peptide Yield Determination

The yield of *H. abdominalis* protein peptides was measured in accordance with our prior published work [26] and calculated by the equation below:

Peptides yields (%) = peptides in the hydrolysate/total protein content of *H. abdominalis* protein × 100 (1)

### 2.6. Measurement of Antioxidant Activities

The free radical scavenging capabilities against DPPH and ABTS were evaluated following our previous protocol [27]. In brief, prepared sample solutions were separately combined with DPPH reagent or ABTS^+^ working solution, and the corresponding absorbance values were recorded at 517 nm and 729 nm, respectively. The following equation was used:

DPPH/ABTS radicals scavenging capacity (%) = (A blank − A sample)/A blank × 100(2)

The scavenging capacity against hydroxyl radicals was additionally determined in this study [28]. A 0.1 M sodium phosphate buffer solution at pH 7.4 was used to dissolve the peptide specimens. Briefly, 1.0 mL of peptide solution was mixed with 0.5 mL of EDTA-FeSO_4_ (2.0 mM), 1.0 mL of phosphate-buffered saline (PBS, pH 7.4), 1.0 mL of safranine T solution, and 1.0 mL of H_2_O_2_ solution. For the control group, H_2_O_2_ was replaced with an equal volume of PBS. After thorough mixing, the reaction mixture was incubated for 60 min at 37 °C to measure absorbance at 520 nm. The calculation was performed in accordance with the formula given below:

Hydroxyl radical scavenging activity (%) = (Acontrol − Ablank)/(Asample − Ablank) × 100(3)

The reducing power was assessed following our previous method [27].

All experiments were performed in three independent replicates.

### 2.7. Evaluation of Stability of HAPH-1 Against Temperature, pH, and Gastrointestinal Digestion

As referenced in our previous method [27]. HAPH-1 (5 mg/mL) was incubated in a water bath across 20–100 °C for 2 h. Unheated HAPH-1 was employed as a control.

To assess pH stability, HAPH-1 solutions were adjusted to pH 3.0–11.0 and incubated for 2 h. After neutralization to pH 7, DPPH scavenging activity was measured.

For stability under gastrointestinal digestion (GID), an equal volume of simulated gastric fluid (containing 1 g/100 mL pepsin, pH 2.0) was mixed with HAPH-1 solution at 5 mg/mL, and the mixture was incubated at 37 °C for 2 h in a shaking incubator. Aliquots of the reaction solution were collected at 30 min intervals to assess DPPH radical scavenging activity.

After completion of the simulated gastric digestion stage, the solution pH was adjusted to 7.5 with 1 mol/L sodium hydroxide. Trypsin was introduced at an E/S of 1:25, and the blended sample was incubated at 37 °C for 2 h. The subsequent experimental procedures were consistent with those applied in the gastric digestion step.

All Experiments were performed in three independent replicates.

### 2.8. Animal Experimental Procedures

The experiment was approved by the Ethics Committee of Medical College of Qingdao University (No. QDU-AEC-2025820). Male ICR mice aged 6–8 weeks were obtained from SPF Biotechnology Co., Ltd., (Beijing, China) (SCXK (Jing) 2024-0001) and raised under standard conditions (temperature 22 ± 2 °C, 12 h light/12 h dark cycle, relative humidity 50–60%). Following one week of acclimatization, stratified randomisation by body weight was applied for group allocation, and the animals were assigned into 4 groups (*n* = 10 per group; 40 in total): (1) Normal control group (NC), (2) Model group (M), (3) *H. abdominalis* peptides group (HAPH-1), orally gavaged with 400 mg/kg/d HAPH-1, (4) 17α-Methyltestosterone group (MT), orally gavaged with 10 mg/kg/d 17α-Methyltestosterone. All experimental mice were provided with free access to standard pellet feed and drinking water throughout the experiment. With the exception of the NC group administered physiological saline, all other animals received daily intraperitoneal injections of triptolide (TP) at a dosage of 150 µg/kg/d. To reduce potential confounding effects, the order of treatments and measurements, as well as the location of animal cages, was systematically randomized. The mice were subjected to a 12 h fast before being sacrificed.

#### 2.8.1. Sperm Quality Analysis

The methodology of [29] was employed to determine the sperm parameters, with slight modifications. After 21 days of intervention, the mice were fasted for 12 h with free access to water. Subsequently, three mice from each group were randomly selected, anesthetized with isoflurane, and sacrificed by cervical dislocation. Both epididymides were carefully dissected, cleared of adhering fat, and the cauda epididymis from the treated mice was promptly dissected and immersed in 5 mL of pre-warmed normal saline maintained at 37 °C. The tissues were minced with sterile ophthalmic scissors and incubated at 37 °C for 5 min to allow complete sperm release, resulting in a sperm suspension.

To assess sperm motility, 30 μL aliquots of the prepared sperm suspension were mounted onto microscope slides in designated groups, covered with coverslips, and examined under a light microscope at ×100 magnification. Sperm motility was evaluated in accordance with the World Health Organization (WHO) 2010 guidelines [29]. Specifically, sperm motility was classified into four categories: rapid-progressive (a): sperm moving rapidly along a straight trajectory with vigorous forward propulsion; slow-progressive (b): sperm moving at a reduced velocity with slow or non-linear forward progression; non-progressive (c): motile sperm showing circular or random motion without forward displacement; and immotile (d): sperm displaying no movement at all. A total of 200 spermatozoa were assessed per sample, and the motility rate was calculated as (a + b + c)/200 [30].

The sperm suspension was aliquoted and loaded onto a hemocytometer, and sperm cells were counted under the same optical microscope at ×100 magnification.

Sperm morphology was evaluated by smearing 30 µL of the suspension onto a clean slide, air-drying, fixing with methanol for 10 min, staining with eosin for 1 h, and then rinsing with water. Slides were examined under the optical microscope at ×100 magnification, and the number of sperm with abnormal morphology was counted out of 200 sperm per mouse. Sperm morphology was also assessed following WHO guidelines. The most common abnormalities, primarily affecting the head and tail, included banana-shaped, round-headed, double-headed, tail-folding, double-tailed, and hookless forms [30].

#### 2.8.2. Determination of Biochemical Indicators

Levels of testosterone (T), follicle-stimulating hormone (FSH), and luteinizing hormone (LH) in serum, and IL-6 and TNF-α in testicular tissue were evaluated by ELISA kits (Jiangsu Jingmei Biological Technology Co., Ltd., Yancheng, China).

Chemical kits (Jiancheng, Nanjing, China) were utilized to evaluate levels of SOD, GSH-PX, total antioxidant capacity (T-AOC), and MDA in testis. All experiments were performed with seven independent biological replicates.

#### 2.8.3. Histological and Immunohistochemical (IHC) Analysis

Fresh samples of testicular tissue underwent fixation using a 4% paraformaldehyde solution (three biological replicates). H&E staining was subsequently carried out following a previously reported protocol [31].

IHC staining for Keap1, Nrf2, Bax, Bcl-2, and Caspase-3 in testicular tissue was quantified according to previously described methods. All IHC images were analyzed using Image-Pro Plus 6.0 software (IPP) [31]. For the IHC and histology analysis, three mice in each group were assessed. One testicular tissue section was prepared from each animal. Three microscopic fields were randomly captured per section at ×100 magnification and quantitatively analyzed.

The IHC staining was quantified based on the positive staining area within the selected microscopic field. The brown DAB-positive staining area was identified using color-based thresholding in IPP. Consistent image acquisition and analysis criteria were applied to all experimental groups. The percentage of positive staining area was calculated as the ratio of the positive staining area to the total area of the selected field × 100%. Finally, all quantitative data were normalized to the NC group. Primary antibodies used were: Keap1 (GB15847, 1:100), Nrf2 (GB113808, 1:500), Bax (GB154122, 1:500), Bcl-2 (GB154380, 1:100) (all rabbit, Wuhan Servicebio Technology Co., Ltd., Wuhan, China); Caspase-3 (GB12532, 1:1000, mouse, Servicebio). Corresponding S-vision poly-HRP secondary antibodies (G1302 for rabbit, G1301 for mouse; Servicebio, ready-to-use) were applied according to the supplier’s standard workflow.

#### 2.8.4. TUNEL Apoptosis Assay

TUNEL staining was performed on testicular tissues from three biologically independent mice per group following a previously established protocol. The TUNEL (red) and DAPI (blue) fluorescence channels were separated using Fiji (ImageJ 1.54p) software, and threshold-based segmentation was applied to quantify the TUNEL-positive area and DAPI-positive nuclear area. The percentage of TUNEL-positive area was calculated as the ratio of TUNEL-positive area to the total DAPI-positive nuclear area, multiplied by 100%. Consistent image acquisition and analysis criteria were applied to all experimental groups [32].

### 2.9. Identification of Peptides

LC-MS/MS technology was utilized to characterize the amino acid sequences and molecular mass values of the isolated peptides, and the related testing service was provided by Sangon Biotech (Shanghai, China) Co., Ltd. Peptide samples were reconstituted, processed with a 10 kDa ultrafiltration tube, desalted by C18 solid-phase extraction, vacuum-dried using a vacuum centrifugal concentrator (CV100-DNA, JIAIM, Beijing, China), and re-dissolved in 0.1% formic acid prior to chromatographic separation. Peptide separation was carried out on an Easy-nLC 1200 nano-UHPLC system (Thermo Fisher Scientific, Waltham, MA, USA). Samples were trapped on a RP-C18 trap column (100 μm × 20 mm, Agilent, Santa Clara, CA, USA) and separated on a PepMap C18 analytical column (75 μm × 25 cm, 2 μm particle size, Thermo Fisher Scientific) at a flow rate of 300 nL/min. Mobile phase A was 0.1% formic acid in water; mobile phase B was 0.1% formic acid in acetonitrile. The linear gradient was programmed as: 5–28% B over 48 min, 28–40% B within 2 min, 40–100% B within 2 min, and held at 100% B until 60 min. Mass spectrometric detection was performed on a Q Exactive Plus mass spectrometer (Thermo Scientific) operated under data-dependent acquisition (DDA) mode. The MS1 full scan covered an *m*/*z* range of 300–1500, with resolution of 70,000 at *m*/*z* 200, AGC target 3 × 10^6^, and a maximum injection time of 100 ms. Following each full-scan acquisition, the top 20 most abundant precursor ions were selected for HCD fragmentation at a normalized collision energy (NCE) of 28. MS2 spectra were acquired at a resolution of 17,500 at *m*/*z* 200, AGC target 1 × 10^5^, maximum injection time 50 ms, with dynamic exclusion set to 25 s.

The raw mass spectrometric data were processed and analyzed using PEAKS Studio 13 (Bioinformatics Solutions Inc., Waterloo, ON, Canada). Peptide identification was performed using database searching. The acquired MS/MS spectra were searched against the UniProt reference proteome database (proteome accession: UP000264820; accessed on 15 August 2025). The database contained 27,735 entries in UniProtKB and was accessed on 15 August 2025. Peptide-spectrum matches with a −10lgP score > 20 were retained as confidently identified peptides. The search parameters were set as follows: a precursor ion mass tolerance of 10 ppm and a fragment ion mass tolerance of 0.03 Da. Acetylation (Protein N-term), Oxidation (M), and Deamidation (NQ) were selected as variable modifications. No missed cleavages or static modifications were specified.

### 2.10. In Silico Screening of Antioxidant Peptides

The prospective bioactivity of identified peptides was evaluated via the PeptideRanker server, applying a threshold score of ≥0.8 as the selection criterion. Physicochemical properties, including pI and GRAVY, were subsequently estimated using the ProtParam tool. Predictions of toxicity and allergenicity were performed using ToxinPred and AllerTOP v2.0, respectively.

### 2.11. Molecular Docking

The two- and three-dimensional configurations of the antioxidant peptides were separately built with ChemDraw 22.00 and Chem3D Ultra 22.0.0.22 programs, after which energy optimization was implemented via the MM2 force field, and the structures were stored as PDB files. The 3D conformations of DPPH (CID: 2735032) and ABTS (CID: 5360881) were acquired from the PubChem database, whereas the structure of Keap1 (PDB ID: 4L7B) was obtained from the RCSB Protein Data Bank. Before molecular docking analysis, the initial ligands in the Keap1 structure were removed using PyMOL 2.6.0a0 software.

When performing docking simulations with Keap1, the target peptides were set as ligands while Keap1 was treated as the receptor. Conversely, for docking simulations performed on DPPH and ABTS radicals, the peptides were set as receptors and the corresponding radicals as ligands. All receptor structures were loaded into AutoDockTools 1.5.7 (ADT), where the following preprocessing steps were carried out sequentially: polar hydrogen addition, Gasteiger charge calculation, and water molecule elimination. Molecular docking was performed using the AutoDock Vina program to predict the optimal binding conformations. Finally, the docking results were visualized and analyzed using PyMOL 2.6.0a0.

### 2.12. Peptides Synthesis

Peptides selected via preliminary screening were prepared through chemical synthesis by Sangon Biotech Co., Ltd. (Shanghai, China), and all products exhibited a purity of 95%.

### 2.13. Statistical Analysis

Statistical analyses were performed using one-way analysis of variance (ANOVA). The homogeneity of variances was assessed before post hoc multiple comparisons. Tukey’s test was used when the assumption of homogeneity of variances was satisfied, whereas Dunnett’s T3 test was applied when this assumption was violated. Statistical analyses and graphical presentations were performed using SPSS 27.0 and GraphPad Prism 9.0, respectively. All data are presented as the mean ± standard deviation (SD), and *p* < 0.05 is considered statistically significant.

## 3. Results

### 3.1. Process Optimization for HAPH Preparation

#### 3.1.1. Selection of Pretreatments

**Figure 1:**
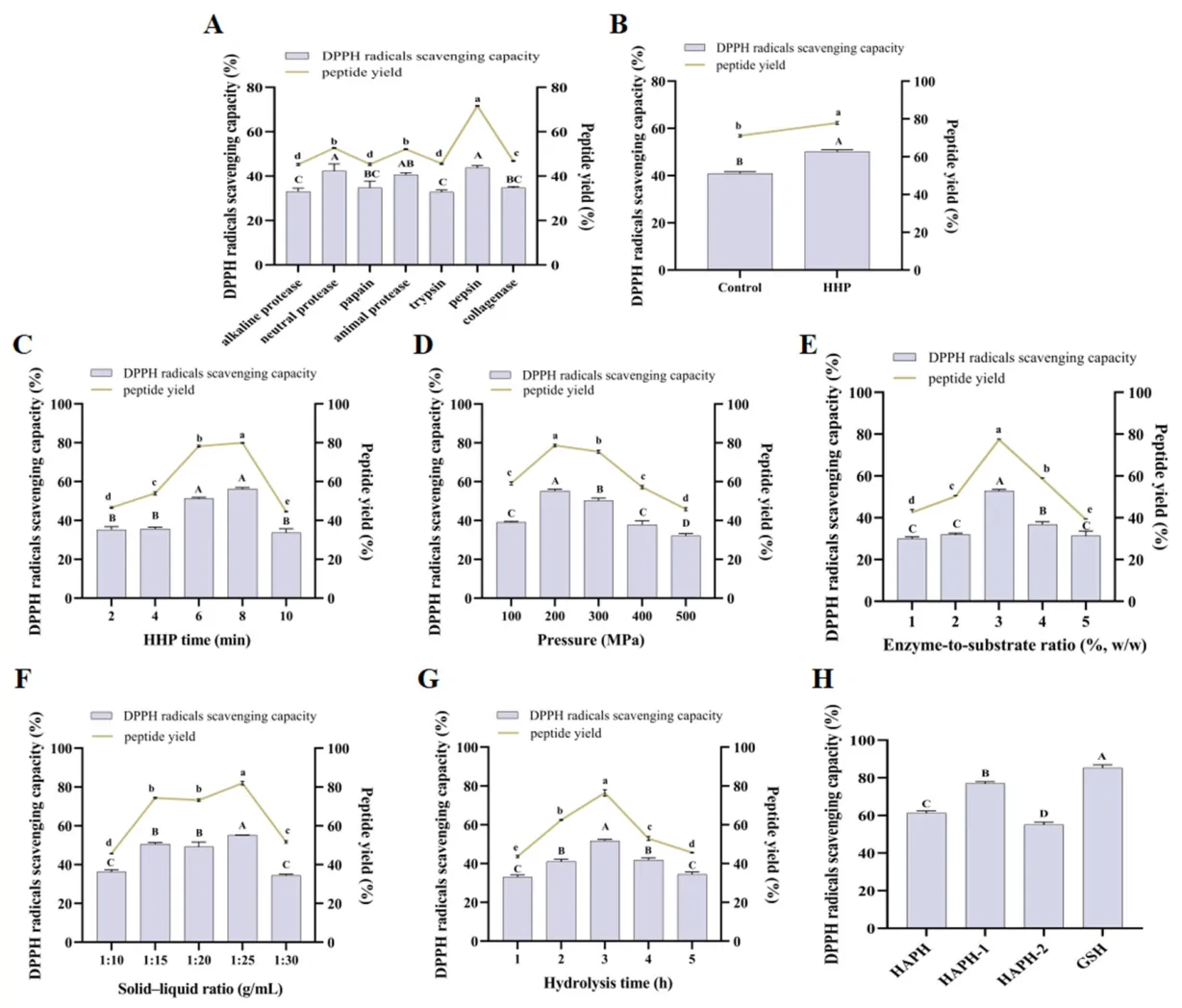
Process optimization and ultrafiltration of *H.abdominalis* protein hydrolysates (HAPH). (**A**) Results for different proteases, (**B**) Results for pretreatment evaluation, (**C**–**G**) Results for different HHP time, HHP pressure, E/S, S/L, hydrolysis time, (**H**) DPPH radical scavenging capacity for each component of ultrafiltration. Data are presented as mean ± SD (*n* = 3). A–D, different capital letters indicate significant differences in DPPH radical scavenging capacity between groups (*p* < 0.05). a–e, different lowercase letters indicate statistical differences in peptide yields between groups (*p* < 0.05).

Seven distinct proteases were employed to hydrolyze *H. abdominalis*, as presented in Figure 1A. Among the seven proteases, the hydrolysate of pepsin demonstrated the highest DPPH radical scavenging capacity (43.77 ± 1.04%) and peptide yield (71.66 ± 0.2%), which were superior to the other enzymatic hydrolysates (*p* < 0.05). Consequently, pepsin was selected for subsequent experiments. Subsequently, HHP treatment was applied to assist pepsin hydrolysis, as shown in Figure 1B. HHP treatment increased the DPPH radical scavenging capacity and peptide yield. Moreover, the hydrolysate produced by HHP pretreatment demonstrated significantly greater DPPH radical scavenging activity and peptide yield (50.08 ± 0.86% and 77.83 ± 0.7%, respectively) than those of the other treatment groups (*p* < 0.05). Therefore, HHP was determined as the optimal pretreatment method.

#### 3.1.2. Single-Factor Experiment

As displayed in Figure 1C, both the DPPH radical scavenging activity and peptide yield peaked at an HHP time of 8 min, with values of 56.23 ± 0.72% and 79.93 ± 0.23%, respectively.

Figure 1D demonstrated that DPPH radical scavenging capacity and peptide yield exhibited significant enhancement (*p* < 0.05) as HHP pressure increased from 100 MPa to 200 MPa. At 200 MPa, the values reached 53.63 ± 0.93% and 78.67 ± 0.67%, respectively. As shown in Figure 1E, an increase in the E/S ratio from 1% to 3% resulted in a significant enhancement in both DPPH radical scavenging capacity and peptide yield. When E/S achieved 3%, the values reached 52.90 ± 0.61% and 77.37 ± 0.23%, respectively. The influence of S/L on these two dependent variables was shown in Figure 1F. At a ratio of 1:25, the *H. abdominalis* achieved maximum DPPH radical scavenging capacity (55.10 ± 0.17%) and peptide yield (81.97 ± 0.90%). With prolonged hydrolysis time (Figure 1G), both parameters gradually increased from 1 to 3 h, reaching peak values of DPPH radical scavenging capacity (51.67 ± 0.83%) and peptide yield (76.43 ± 1.62%) at 3 h.

#### 3.1.3. Plackett–Burman and Response Surface Experiments

Plackett–Burman experimental design was employed to screen the five factors (E/S, enzymatic hydrolysis time, S/L, HHP pressure, and HHP time) for their influence on the comprehensive score of peptide yield and DPPH radical scavenging capacity.

As presented in Table S1, the regression models for these two response variables were statistically significant (*p* < 0.05), confirming the reliability of the models. The factors influencing peptide yield ranked E > A > C > B > D, while factors affecting DPPH radical scavenging activity showed a descending order of A > C > E > D > B. Among them, factors A, C, and E showed significant effects on peptide yield (*p* < 0.05), while factors A and C significantly influenced the DPPH radical scavenging capacity (*p* < 0.05). Therefore, E/S (A), S/L (C), and HHP time (E) were selected as the three factors for the Box–Behnken experimental design.

The Box–Behnken experimental design was implemented with E/S (A’), S/L (B’), and HHP time (C’) as independent variables, while peptide yield (Y1) and DPPH radical scavenging capacity (Y2) served as response variables. Multiple regression analysis was performed on the data outlined in Table S2, generating the following predictive equations:

Y1 = 81.8 − 0.194A − 0.305B − 0.796C − 0.305AB − 0.412AC + 0.74BC − 6.42A^2^5.96B^2^ − 5.79C^2^

Y2 = 60.2 − 0.48A + 0.144B − 0.496C + 0.0275AB − 0.453AC + 0.415BC − 3.49A^2^ − 2.67B^2^ − 3.28C^2^

**Figure 2:**
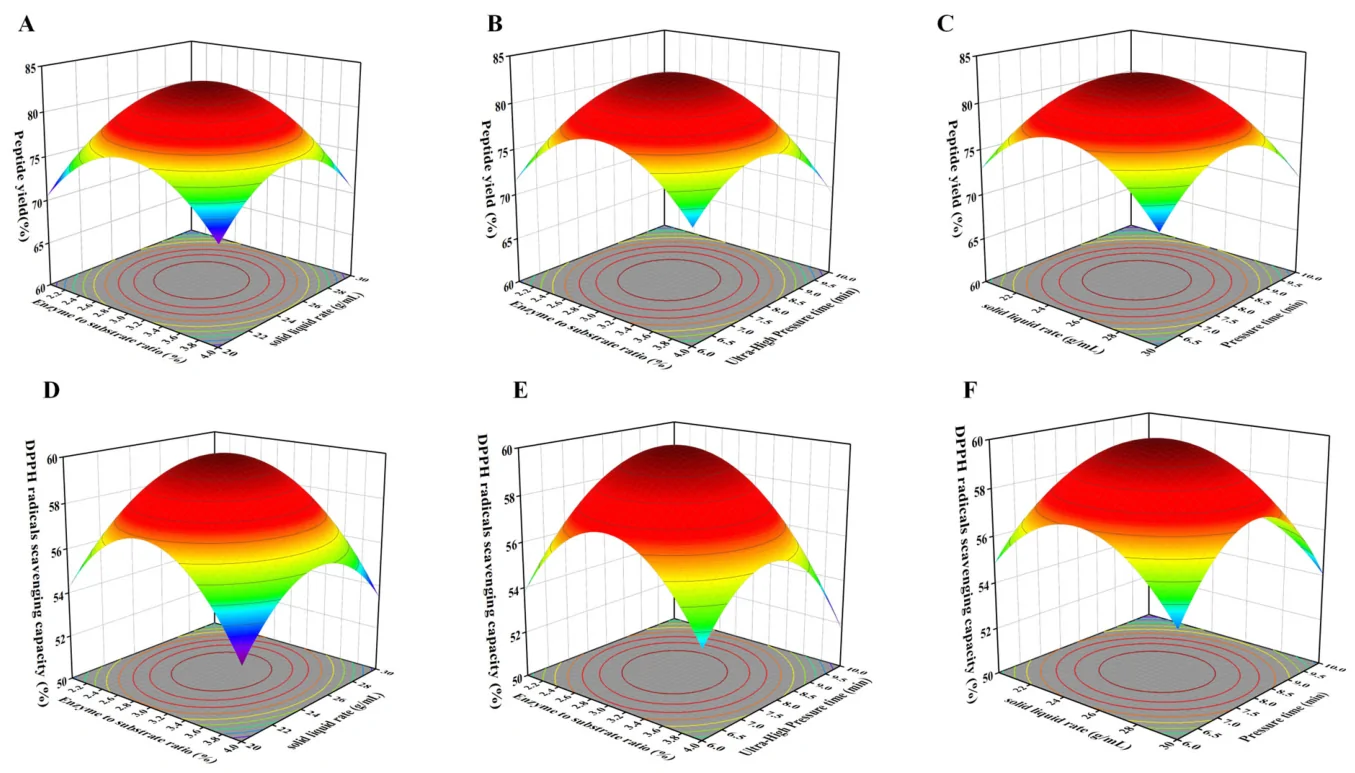
Results of the response surface experiment. (**A**) Response surface effects of E/S and S/L on the peptide yield, (**B**) Response surface the effects of E/S and HHP time on the peptide yield, (**C**) Response surface the effects of S/L and HHP time on the peptide yield, (**D**) Response surface effects of E/S and S/L on DPPH radicals scavenging capacity, (**E**) Response surface the effects of E/S and HHP time on DPPH radicals scavenging capacity, (**F**) Response surface the effects of S/L and HHP time on DPPH radicals scavenging capacity. The color gradient from red to blue represents the gradual decrease of response values (peptide yield or DPPH radical scavenging capacity).

As shown in Table 1 and Figure 2, the quadratic terms of E/S (A’^2^), S/L (B’^2^), and HHP time (C’^2^) all exhibited significant effects on peptide yield and DPPH radical scavenging activity. C’ and the interaction term of S/L and HHP time (B’C’) show a significant impact on peptide yield (*p* < 0.05), while A’, C’, and the interaction term of E/S and HHP time (A’C’), as well as S/L and HHP time (B’C’), have a significant effect on DPPH radical scavenging activity (*p* < 0.01). Both regression models demonstrated highly significant validity (*p* < 0.01). The lack-of-fit terms for the models were non-significant, with coefficients of determination (R^2^) of 0.9949 and 0.9968 for peptide yield and DPPH radical scavenging activity, respectively. All coefficients of variation (CV) were below 5%.

Optimal conditions derived from Box–Behnken analysis were determined as follows: 1:25.051 for S/L, 2.97% for E/S, and 9.12 min for HHP time, yielding theoretical peptide yield and DPPH radical scavenging activity of 79.55% and 58.93%, respectively. For practical implementation, the parameters were adjusted to E/S of 3%, hydrolysis time of 3 h, S/L of 1:25, HHP pressure of 200 MPa, and 9 min of HHP time. Under these optimized conditions, the hydrolysate exhibited a peptide yield of 82.69% and DPPH radical scavenging activity of 60.97%, showing no significant difference between actual and predicted values (*p* > 0.05).

### 3.2. Stability for HAPH Fraction with Best DPPH Radical Scavenging Activity

The DPPH radical scavenging activities of different HAPH fractions obtained by ultrafiltration were shown in Figure 1H. Among them, HAPH-1 exhibited the strongest activity compared with HAPH and HAPH-2 (*p* < 0.05). Consequently, HAPH-1 was chosen for subsequent research.

**Figure 3:**
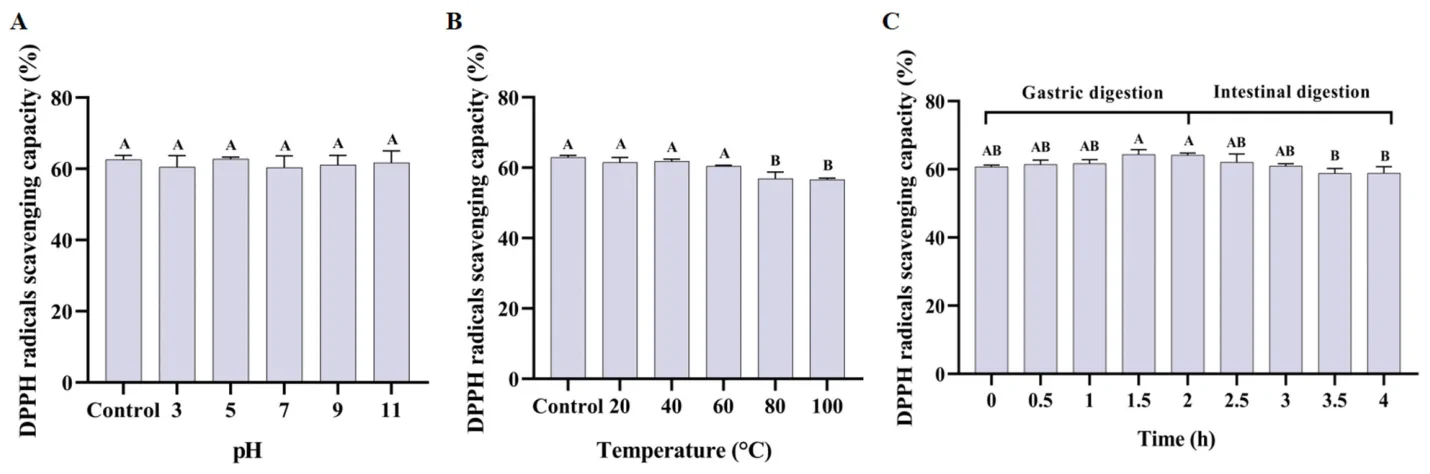
Results of the in vitro stability assessment for the HAPH-1 fraction exhibiting the highest antioxidant activity. (**A**) pH stability, (**B**) Thermal stability, and (**C**) gastrointestinal stability. Data are presented as mean ± SD (*n* = 3). A–B indicate significant differences in DPPH radical scavenging capacity between groups (*p* < 0.05).

In vitro assessments of HAPH-1 stability toward pH, thermal stress, and simulated gastrointestinal digestion are depicted in Figure 3A–C. No obvious variation in the DPPH radical scavenging ability of HAPH-1 was observed at pH ranging from 3 to 11 and temperatures of 20 to 60 °C in comparison with the control (*p* > 0.05). At temperatures of 80 to 100 °C, a slight decline in the ability to scavenge DPPH radicals was observed, reaching values of 56.90 ± 1.87% and 56.60 ± 0.44% (*p* < 0.05).

### 3.3. Effect of HAPH-1 on Growth Parameters in Male Mice with TP-Induced Reproductive Injury

**Figure 4:**
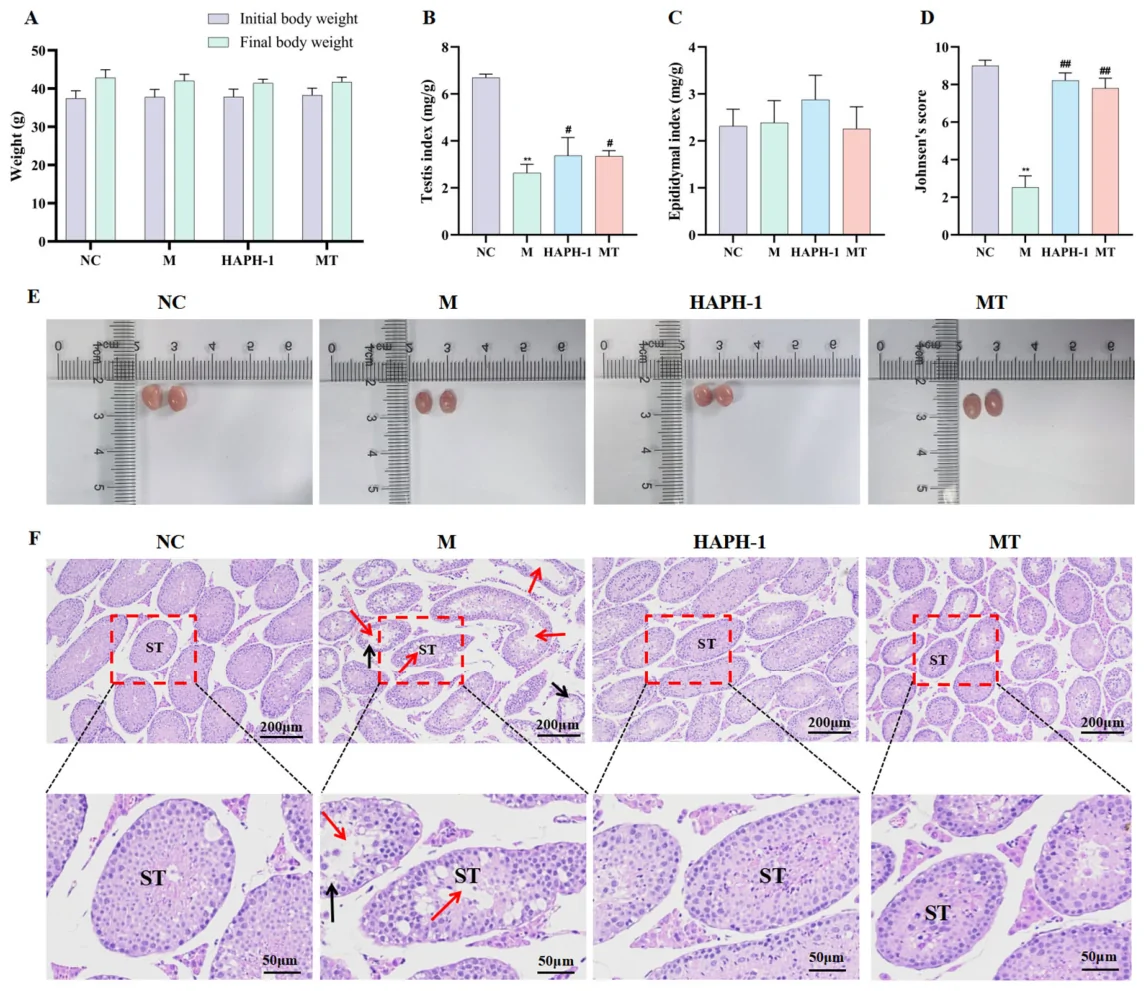
Effects of HAPH-1 on growth parameters in mice. (**A**) Mouse body weight, *n* = 10 for initial body weight and n = 7 for final body weight; (**B**,**C**) Testicular index and epididymal index in mice (*n* = 7); (**D**) Johnsen’s score (*n* = 7), (**E**) Testicular morphology and (**F**) HE-stained section in mice (red boxes indicate the magnified regions) (*n* = 3). ST indicates the seminiferous tubules; Black arrow indicates wide spaces between these spermatogenic cells; Red arrow indicates wide lumen of the tubule; Data are presented as mean ± SD. ** *p* < 0.01 versus NC group; # *p* < 0.05, ## *p* < 0.01 versus M group.

As shown in Figure 4A,C, no statistically significant variations were detected in initial body weight, final body weight, and epididymal index across all experimental groups (*p* > 0.05). However, compared with the NC group, the testicular index (Figure 4B) was significantly lower in the M group (*p* < 0.01). Moreover, the testes exhibited marked atrophy, a dull and dusky purple coloration, and obvious shrinkage of the tunica albuginea. Following HAPH-1 administration, the testicular index showed a significant increase (*p* < 0.05), along with a notable recovery in testicular volume, brighter coloration, and a more intact tunica albuginea.

Spermatogenesis and sperm quality are closely dependent on the structure of testicular tissue and the count of testicular cells. H&E staining provided a clear visualization of testicular damage in each group. Figure 4E showed that the testicular structure of the NC group appeared normal, with an intact epithelial membrane and closely arranged seminiferous tubules. However, after exposure to TP, the testis tissue of the M group presented a significantly increased number of regressed seminiferous tubules, exfoliation of germ cells, disorganization of the germinal epithelium, appearance of blank cavities in the testis, and atrophied seminiferous tubules with only a few Sertoli cells, spermatogonia, and primary spermatocytes. These phenomena suggested severe destruction of the testicular structure in the M group. Following intervention with HAPH-1, well-expanded luminal diameters were observed, and lumina were densely packed with germ cells at various developmental stages in a tightly organized arrangement; tissue vacuolization was markedly reduced. The morphological structure of the testes was examined in each animal, and Johnsen’s score (Figure 4D) was evaluated according to spermatogenic function and germ cell numbers, with a scoring range from 1 (lack of spermatogenic epithelium) to 10 (regular spermatogenesis) [33]. Compared with the M group, HAPH-1 significantly increased the declined Johnsen score in TP-treated mice (*p* < 0.01). These findings suggest that HAPH-1 reversed the morphological abnormalities in the testicular tissue of mice.

### 3.4. Effects of HAPH-1 on Sperm Quality of TP-Induced Mice

**Figure 5:**
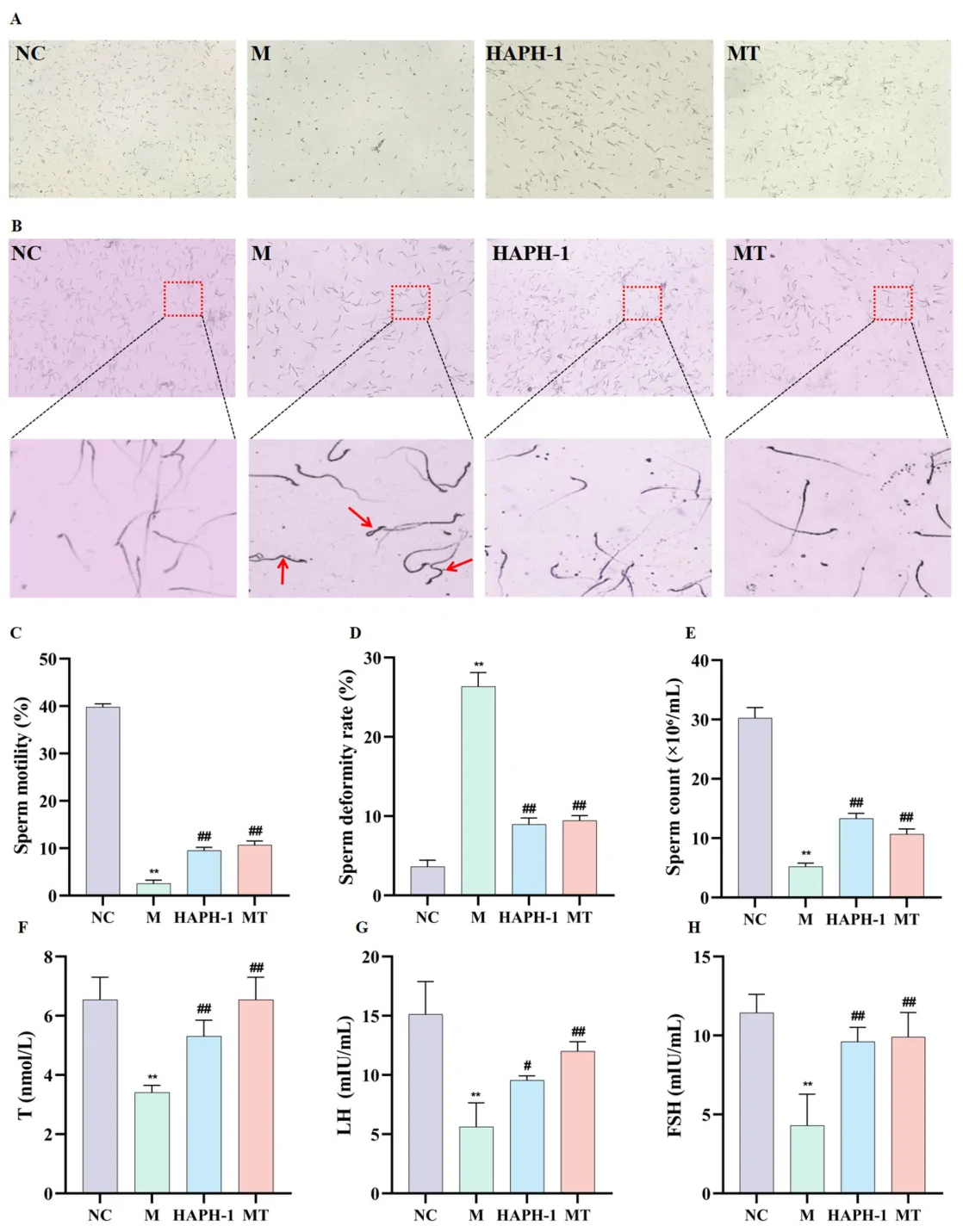
Effects of HAPH-1 on sperm quality of mice induced by TP. (**A**) Sperm number was assessed. (**B**) Sperm morphology was examined. Representative images were captured at ×100 magnification using a light microscope (red boxes indicate the magnified regions) (*n* = 3). Red arrow: abnormal spermatozoa. (**C**–**E**) Indicate the sperm motility, count, and deformity rate of sperm, respectively (*n* = 3); (**F**–**H**) T, LH, and FSH levels, respectively (*n* = 7). Data are presented as mean ± SD. ** *p* < 0.01 versus NC group; # *p* < 0.05, ## *p* < 0.01 versus M group.

Sperm quality serves as a critical parameter for evaluating male reproductive function, with sperm motility, sperm count, and sperm deformity rate being the main parameters. After 21 days of TP exposure, Sperm quality was assessed using an optical microscope. As shown in Figure 5A–D, compared with the NC group, the M group showed a significant decrease in sperm motility and sperm count (*p* < 0.01), along with a significant increase in sperm deformity rate (*p* < 0.01). Abnormal sperm morphology, including head deformities and folded bodies, was observed in the M group. Subsequently, as displayed in Figure 5E–G, we examined sex hormone levels in mice and found that exposure to TP caused a marked decrease in serum levels of T, LH, and FSH compared to the NC group (*p* < 0.01), indicating that TP disrupted serum reproductive hormone levels in male mice. Intervention with HAPH-1 significantly restored sperm motility, sperm count, and sex hormone levels (*p* < 0.05), while also reducing the sperm deformity rate (*p* < 0.01).

### 3.5. HAPH-1 Reduced Oxidative Stress in Testis of TP-Treated Mice

**Figure 6:**
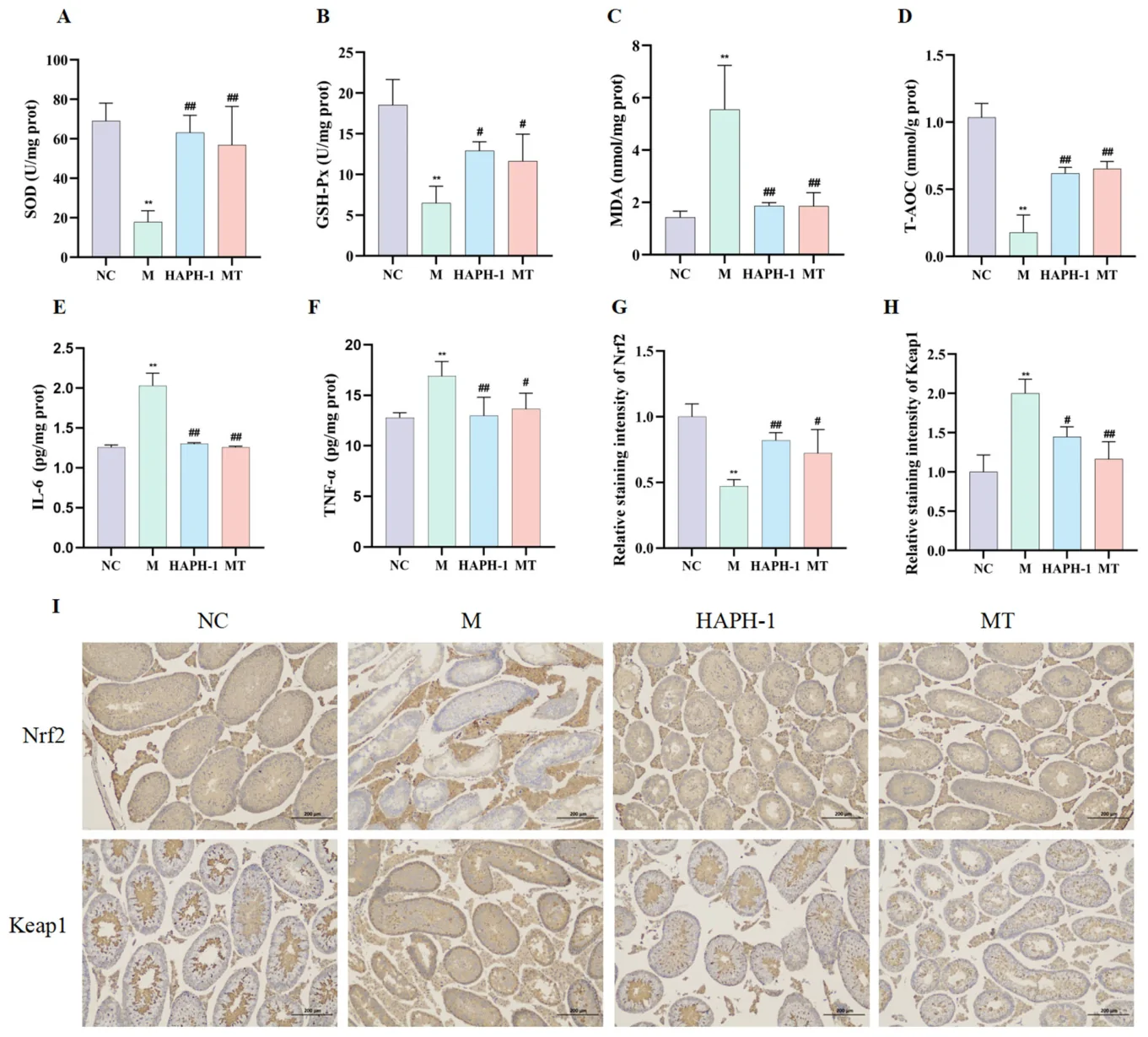
Effects of HAPH-1 on oxidative stress and inflammatory factor-related indices in testicular tissue. (**A**–**D**) Testes SOD, GSH-PX, MDA, T-AOC levels (*n* = 7), (**E**,**F**) Testes IL-6, TNF-α levels (*n* = 7), (**G**,**H**) Relative expression levels for total Nrf2, Keap1 in testicular tissue (*n* = 3), (**I**) Immunohistochemical results (100×) of Keap1, Nrf2 in testicular tissue. Data are presented as mean ± SD. ** *p* < 0.01 versus NC group; # *p* < 0.05, ## *p* < 0.01 versus M group.

Testicular damage is typically associated with oxidative stress. As illustrated in Figure 6A–D, compared with the NC group, TP administration notably increased testicular MDA levels while lowering the activities of SOD, GSH-PX, and T-AOC (*p* < 0.01). HAPH-1 group had lower testicular levels of MDA (*p* < 0.05) and higher levels of SOD, GSH-PX, and T-AOC than the M group (*p* < 0.01). Furthermore, inflammatory signaling pathways were triggered under conditions of oxidative stress. Therefore, we measured the concentrations of cytokines related to testicular inflammation. As shown in Figure 6E,F, in the M group, the concentrations of IL-6 and TNF-α within the testes were significantly raised; however, treatment with either HAPH-1 substantially reduced these elevated levels (*p* < 0.01). In addition, immunohistochemical staining was employed to assess the protein expression of Nrf2 and Keap1 in testicular tissue (Figure 6G–I), which are key members of the Nrf2 pathway that is closely linked to oxidative stress. According to the findings, the M group exhibited increased relative staining intensity for Keap1 and decreased intensity of Nrf2 relative to the NC group (*p* < 0.05). Following treatment with HAPH-1, the relative staining intensity of Keap1 was reduced (*p* < 0.05), whereas that of Nrf2 was notably increased (*p* < 0.05).

### 3.6. Effects of HAPH-1 on Testicular Apoptosis Induced by TP

**Figure 7:**
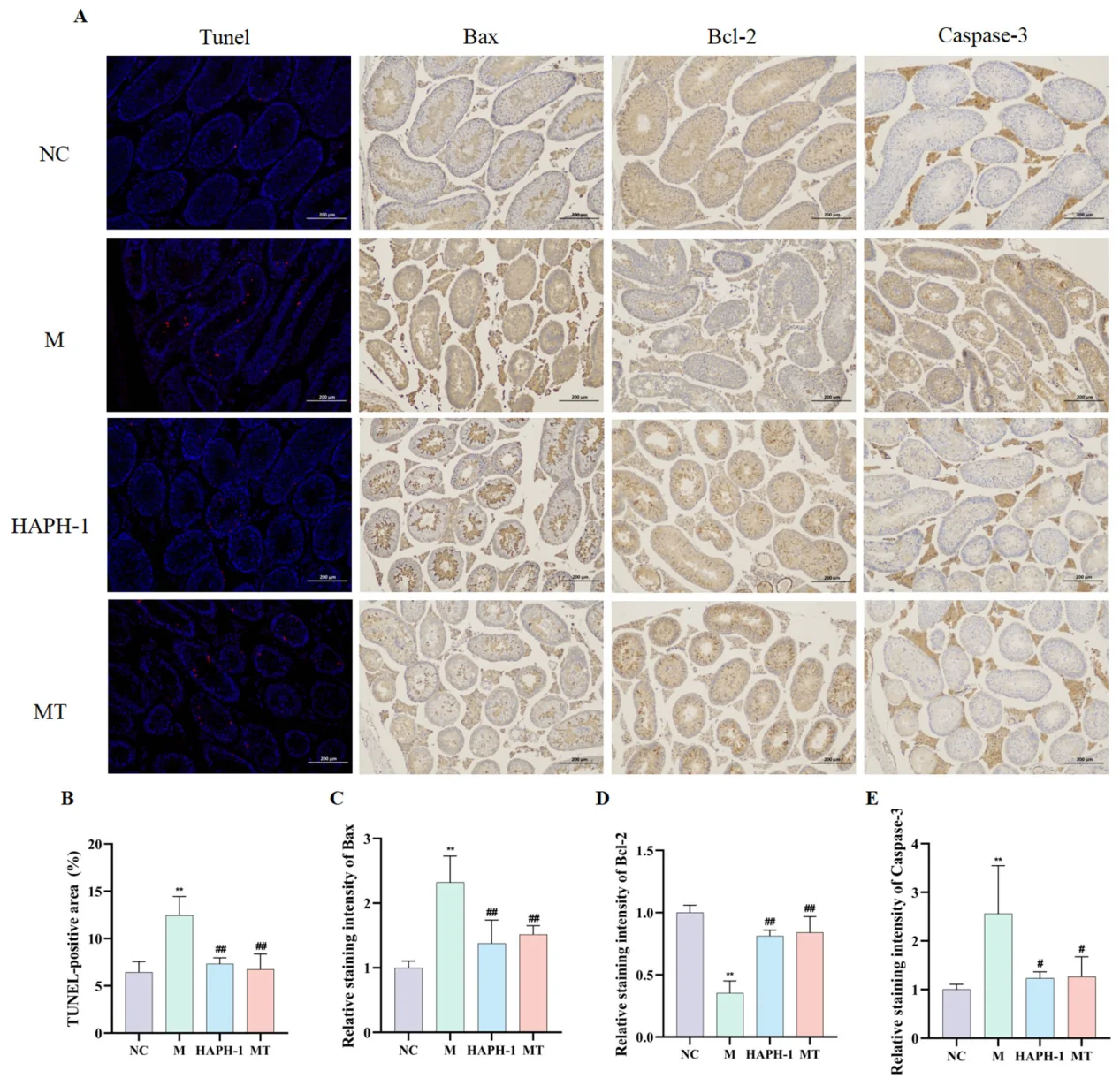
Effects of HAPH-1 on apoptotic index in testicular tissue of mice induced by TP. (**A**) Immunohistochemical results (100×) and apoptotic cells in testicular tissue sections were detected by TUNEL assay (apoptotic cells: red fluorescence, cell nucleus: blue fluorescence); (**B**–**E**) The ratios of apoptotic cells were shown, and the relative expression levels of Bax, Bcl-2, and Caspase-3 in testicular tissue, respectively. Data are presented as mean ± SD (*n* = 3).** *p* < 0.01 versus NC group; # *p* < 0.05, ## *p* < 0.01 versus M group.

We investigated cell apoptosis in testes of mice. Figure 7A displays the results of TUNEL staining, which was employed to assess cell apoptosis. In the testicular tissue collected from the control group, only a limited number of apoptotic cells were observed. By contrast, the TP group exhibited a marked rise in the percentage of apoptotic cells, as illustrated in Figure 7B (*p* < 0.01). After intervention with HAPH-1, the area of apoptotic cells showed a substantial reduction (*p* < 0.05). To further elucidate the causes of testicular cell apoptosis, the protein expression of Bcl-2, Bax, and caspase-3 markers was analyzed. As shown in Figure 7C–E, treatment with TP led to higher relative staining intensities of both Bax and caspase-3 (*p* < 0.05), and inhibited that of Bcl-2 (*p* < 0.01). The relative staining intensities of Bax and caspase-3 were notably suppressed, whereas the intensity of Bcl-2 was increased after HAPH-1 treatment (*p* < 0.05). These results demonstrated that HAPH-1 could alleviate apoptosis in testicular tissue caused by TP exposure.

### 3.7. Screening and Identification of Bioactive Peptides

LC-MS/MS analysis was conducted to determine the peptide sequences present in HAPH-1. A total of 4033 peptides with molecular weights < 3 kDa and peptide lengths of 6–33 amino acids were identified. Detailed information on all identified peptide sequences is provided in Supplementary Data S1. Subsequently, bioinformatics analysis was performed to predict the activity of the identified peptides. The non-toxic and non-allergenic top 50 peptides with the highest bioactivity scores are shown in Table 2. These 50 peptides were designated as P1–P50.

### 3.8. Results of Molecular Docking

Lower binding energy indicates more stable and thermodynamically favorable interactions between peptides and ligands. Results from molecular docking with Keap1 showed that the binding energies of GPAPWGF (P1), FPALLF (P2), and LPLPLF (P4) were the lowest among the 50 peptides and were all less than −9 cal/mol (Table 2).

**Figure 8:**
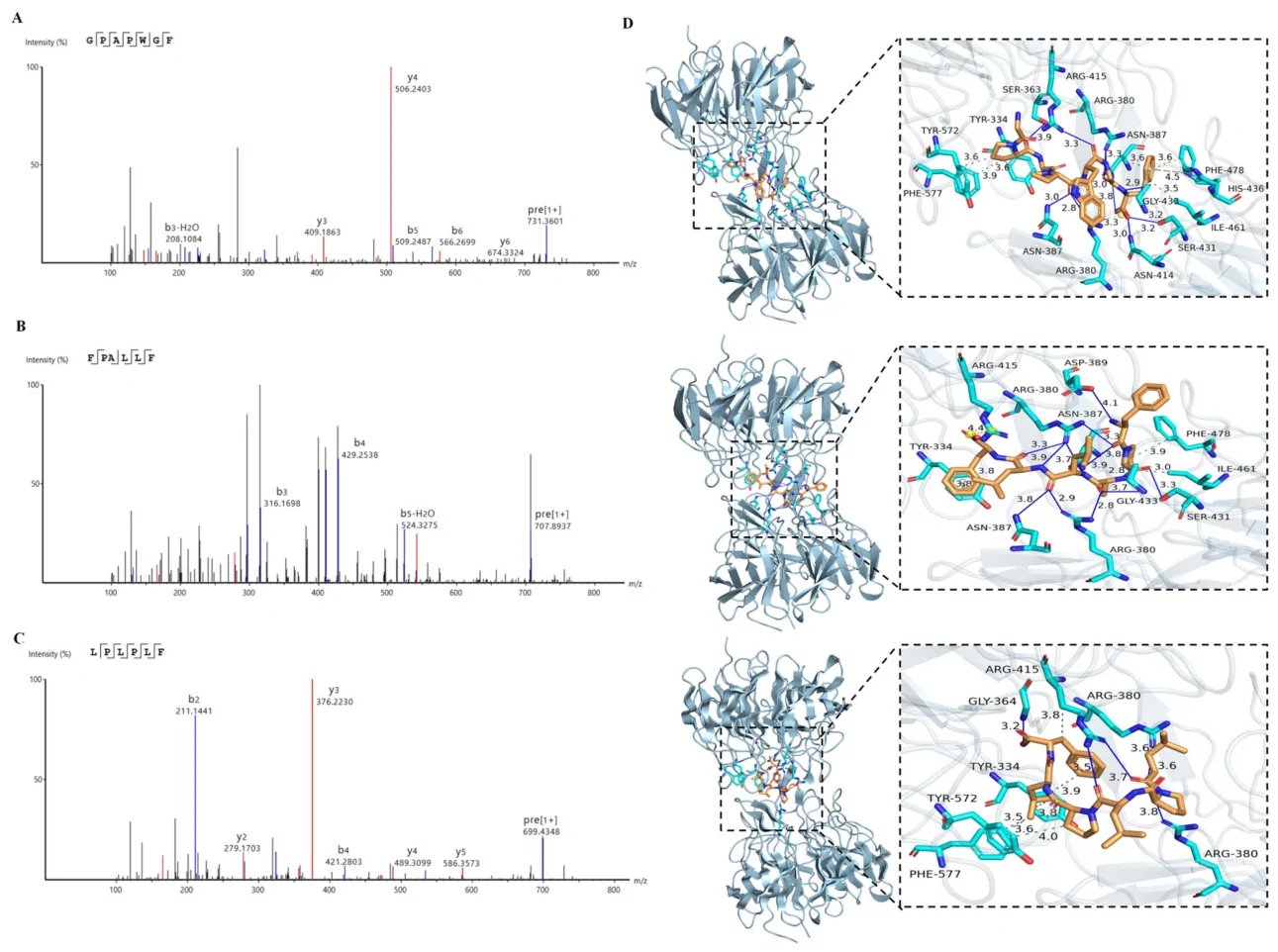
Identification of novel peptides from HAPH-1. (**A**–**C**) P1, P2, and P4 secondary mass spectra. Black peaks are experimentally detected fragment ions. Red vertical lines represent theoretical b-ions, and blue vertical lines represent theoretical y-ions.; (**D**) Molecular docking results of peptides with Keap1. Peptides are shown in orange, and residues of Keap1 are presented in blue. Blue solid lines represent hydrogen bonds, and dashed lines represent hydrophobic interactions.

Molecular docking of the three peptides with DPPH and ABTS radicals yielded relatively low binding energies (Table S4). The actual antioxidant capacity was ultimately validated by the in vitro chemical assays (Table 3). Accordingly, P1, P2, and P4 were inferred to exhibit potent antioxidant capacity. These three peptides were thus chosen for result visualization and analysis (Figure 8B), with their interaction forces with Keap1 detailed in Table S3.

### 3.9. Antioxidant Activity of Synthetic Peptides

Collective data from molecular docking analysis implied that peptides P1, P2, and P4 possessed notable potential antioxidant activity, and their novel sequences were verified via the BIOPEP-UWM database as well as manual retrieval. Accordingly, these three peptides were chemically synthesized in vitro for further validation of their antioxidant effects. The results (Table 3) indicated that, compared to HAPH-1. The DPPH IC_50_ values of P1, P2, and P4 were significantly lower than those of HAPH-1 (*p* < 0.05), which were 1.48 ± 0.13 mg/mL, 2.32 ± 0.18 mg/mL, and 2.21 ± 0.18 mg/mL, respectively. Similarly, the ABTS radical IC_50_ values of P1, P2, and P4 were also markedly decreased compared with HAPH-1 (*p* < 0.05), recorded as 0.39 ± 0.01 mg/mL, 0.42 ± 0.03 mg/mL, and 0.56 ± 0.02 mg/mL. In addition, the reducing power of P1, P2, and P4 was significantly higher than that of HAPH-1 (*p* < 0.05). The antioxidant properties of the synthesized peptides were highly consistent with the molecular docking results.

## 4. Discussion

Male reproductive dysfunction is not only a major cause of infertility, but also associated with increased incidence of numerous diseases [34]. In this study, antioxidant peptides were prepared from *H. abdominalis* through enzymatic hydrolysis optimization, and the optimal processing condition was determined as HHP time 9 min, HHP pressure 200 (Mpa), E/S of 3% (*w*/*w*), S/L 1:25 (*v*/*w*), pH of 2, temperature of 37 °C, and enzymatic time of 3 h. The enzymatic hydrolysate prepared under optimal enzymatic conditions (HAPH) was fractionated using a 3 kDa ultrafiltration membrane to obtain the fraction with molecular weight *<* 3 kDa (HAPH-1). In vitro antioxidant assays demonstrated that HAPH-1 displayed stronger antioxidant activity than other fractions. Meanwhile, HAPH-1 showed excellent thermal, pH, and gastrointestinal digestion stability. In TP-induced reproductive injury male mice, HAPH-1 effectively improved sperm quality, increased testicular index, reduced oxidative stress, and chronic inflammatory status in testicular tissue. The underlying mechanism was related to the regulation of the hypothalamic-pituitary-testis (HPT) axis, the Nrf2-Keap1 antioxidant pathway, and the Bcl-2/Bax/caspase-3 apoptotic signaling pathway. Moreover, three new peptides (GPAPWGF, FPALLF, and LPLPLF) possessing potent antioxidant properties were confirmed and identified by means of LC-MS/MS analysis, molecular docking, and artificial peptide synthesis.

Peptides generated through hydrolysis by diverse proteases exhibit distinct amino acid profiles and structural sequences, leading to varied biological functions. Different proteases possess distinct cleavage sites, thereby generating diverse bioactive peptides. In this study, by considering both the antioxidant bioactivity (DPPH radical scavenging activity) and yield of peptide, we screened the optimal protease and identified pepsin as the best protease. Pepsin, the principal enzyme involved in protein digestion in the gastric phase, efficiently hydrolyzes peptide bonds between hydrophobic amino acids, particularly aromatic residues including tryptophan, tyrosine, and phenylalanine [35]. Jayaprakash et al. [36] reported that peptides obtained from pepsin hydrolysis of New Zealand green-lipped mussel protein exhibited superior scavenging capacities against DPPH and ABTS than those obtained from other proteases. Mazloomi-Kiyapey et al. [37] obtained bioactive peptides with the highest antioxidant activity from medicinal pumpkin seeds protein via pepsin hydrolysis. This evidence supports our choice of pepsin as the optimal enzyme for acquiring HAPH.

As a novel non-thermal processing technology, HHP has found extensive applications in the food industry, mainly due to its favorable characteristics such as low operational expense, excellent safety, rapid processing duration, and negligible nutrient degradation [38]. Recently, increasing evidence has proven the efficiency of HHP in producing bioactive peptides. Under high-pressure treatment, proteins are unfolded, and the internal polar and hydrophobic groups of proteins are exposed, increasing the surface charge distribution and water-binding capacity of protein, thereby improving the solubility of protein and affecting protein hydrolysis [25]. Al-Ruwaih et al. [39] reported that HHP combined with enzymatic treatment of kidney bean protein isolate notably enhanced both the hydrolysis extent and antioxidant performance. Zhu et al. [26] reported that HHP pretreatment prior to enzymatic hydrolysis of sweet potato protein increased both peptide yield and α-glucosidase inhibitory activity. Consistent with the aforementioned studies, our results demonstrated that HHP pretreatment increased peptide yield by 11.77% and DPPH radical scavenging activity by 5.4% compared to single enzymatic hydrolysis. Collectively, these results confirmed that HHP pretreatment was an effective strategy to improve the enzymatic hydrolysis efficiency of *H. abdominalis* protein and enhance the antioxidant potential of its hydrolysates.

Single-factor and response surface methodology experiments are widely utilized strategies for the optimization of the enzymatic hydrolysis process. Here, S/L 1:25, E/S 3%, hydrolysis time of 3 h, HHP time of 9 min, and HHP pressure of 200 MPa were identified as the optimal enzymatic hydrolysis conditions by single-factor and response surface experiments. Under the best condition, the peptide yield and DPPH radical scavenging activity of HAPH reached 82.69% and 60.97%, respectively. The biological activity of peptides is closely associated with their molecular weight, and it is generally recognized that small-molecular-weight peptides possess superior activity [40]. In this study, *H. abdominalis* protein hydrolysates were fractionated, and the fraction with Mw < 3 kDa (HAPH-1) displayed the strongest DPPH radical scavenging activity. The IC_50_ values of HAPH-1 for scavenging DPPH, ABTS, and hydroxyl radicals, as well as its reducing power (2 mg/mL peptide), were 3.043 ± 0.05 mg/mL, 0.763 ± 0.06 mg/mL, 1.259 ± 0.04 mg/mL, and 0.332 ± 0.01, respectively. Chen et al. prepared alkaline protease hydrolysate of *H. abdominalis* and reported its IC_50_ value for DPPH radical scavenging activity as 4.549 mg/mL, which is higher than our results [41]. Kim et al. [19] obtained three fractions (<5 kDa, 5–10 kDa, and >10 kDa) from the enzymatic hydrolysate of *H. abdominalis* via enzymatic hydrolysis and ultrafiltration. Among them, the fraction with MW < 5 kDa exhibited the highest peroxyl radical scavenging activity, with an IC_50_ of 0.46 mg/mL. Wu et al. [28] obtained frog skin peptides with MW below 3 kDa by ultrafiltration and reported that their IC_50_ values for DPPH, ABTS, and hydroxyl radical scavenging activities were 3.3 ± 0.4 mg/mL, 1.9 ± 0.3 mg/mL, and 3.0 ± 0.3 mg/mL, respectively. These results support our findings and prove that HAPH-1 possesses remarkable antioxidant capacity.

The stability of BPs is crucial for exerting their biological functions in vivo and for resisting the complex processing and storage conditions encountered in food production [42]. The stability of HAPH-1 toward high temperature, pH variations, and gastrointestinal digestion was assessed. In this study, HAPH-1 remained stable at pH 3–11 and temperatures of 20–60 °C. This phenomenon can be attributed to the formation of certain secondary structures in low-molecular-weight peptides, which serve as an important structural basis for thermal stability. Temperatures exceeding 60 °C impose mild disturbances on these secondary structures, thereby leading to a slight reduction in antioxidant activity [43]. Compared with the control group, HAPH-1 showed an increase in DPPH radical scavenging activity after 1.5–2 h of pepsin digestion. Enzymes are labile molecules whose activity depends on several concurrent factors, including substrate and enzyme concentrations, as well as reaction conditions such as temperature, pH, and the presence of activators or inhibitors [44]. Therefore, in the simulated gastric digestion system, pepsin can further hydrolyze HAPH-1 under the new enzymatic environment and produce novel bioactive peptides. However, the antioxidant capacity of HAPH-1 slightly declined after further tryptic digestion. Intestinal enzymes target numerous cleavage sites, leading to extensive hydrolysis of the peptides and further degradation of bioactive peptides, thus weakening their antioxidant activity [45]. We proved that HAPH-1 exhibited good stability, indicating its great potential for widespread applications in food processing.

TP, a diterpene epoxide extracted from the roots of *Tripterygium wilfordii* (GTW), has multiple toxicities and side effects, with reproductive toxicity being the most significant and having a high incidence [46]. Thus, TP is frequently employed to build male reproductive injury animal models [47]. In this study, we successfully established a male reproductive disorder mouse model via intraperitoneal injection (i.p.) of TP, which is characterized by decreased sperm quality, damaged testicular structure, disrupted reproductive hormone levels, exacerbated oxidative stress, and chronic inflammation in mice. These observations are consistent with previous studies [33]. In this study, 400 mg/kg (equivalent to 2.4 g/day in humans) HAPH-1 alleviated TP-induced testicular injury, characterized by improved testicular index, morphological structure of testicular tissue, and the number of germ cells in seminiferous tubules. Meanwhile, sperm motility was markedly enhanced by HAPH-1 treatment, characterized by increased sperm count, decreased sperm malformation rate, and enhanced sperm motility. Previous studies have reported the improvement effects of protein hydrolysates from seahorse on male reproductive function in normal mice [20] and diabetic rats [11]. Our study was the first report on the effects of peptides from seahorse on TP-induced male reproductive injury. Zhang et al. [33] reported that oyster peptides (400 mg/kg) significantly alleviated TP-induced reproductive damage in male mice, ameliorating testicular injury and spermatogenic dysfunction. Our results further proved that HAPH-1 is effective in ameliorating male reproductive dysfunction.

The mechanisms underlying TP-induced male reproductive damage mainly include injury to the hypothalamic-pituitary-testicular axis (HPT), exacerbation of oxidative stress, and promotion of apoptosis [48]. Within the HPT axis, the hypothalamus synthesizes and secretes gonadotropin-releasing hormone (GnRH), which in turn promotes the production of FSH and LH from the pituitary gland. These sex hormones coordinately regulate spermatogenesis in the testes [49]. In this study, TP dramatically reduced the serum levels of T, FSH, and LH, suggesting that TP induced abnormal sex hormone secretion [50]. After treatment with HAPH-1, the levels of T, LH, and FSH were dramatically upregulated, suggesting the restoration of sex hormone homeostasis. The results indicated that HAPH-1 can improve reproductive function by regulating the HPT axis. Sudirman et al. [11] found that hydrolyzate of *Hippocampus kuda* improved reproductive dysfunction in diabetic rats by increasing the level of T, FSH, and LH, which is consistent with our results.

Growing evidence has revealed that TP induces oxidative stress in the testis by stimulating the production of extra ROS [51]. Excessive ROS attack and destroy testicular tissue, inducing tissue damage that compromises testicular function and sperm quality [52]. SOD, a highly stable intracellular antioxidant enzyme, functions as an efficient free radical scavenger. GSH engages in the elimination of intracellular reactive oxygen species. MDA, the final product of lipid peroxidation, is widely acknowledged as a crucial biomarker for oxidative stress [53]. Oxidative stress is closely associated with inflammation. Overproduction of ROS stimulates the activation of various pro-inflammatory signaling cascades, which in turn facilitates the secretion of inflammatory cytokines [54]. Herein, HAPH-1 treatment significantly increased levels of SOD, GSH, and T-AOC, while decreasing those of MDA, TNF-α, and IL-6 in testicular tissue of TP-administered mice, indicating the alleviation of TP-induced oxidative stress and chronic inflammation in testicular tissue. The Keap1-Nrf2 signaling pathway serves as a crucial intracellular antioxidant pathway and exerts a vital protective effect on male reproductive health [55]. The transcription factor Nrf2 is extremely sensitive to oxidative stress [56]. Under normal physiological conditions, Nrf2 binds to Keap1 in the cytoplasm and is sequestered therein, thereby inhibiting its nuclear translocation and downstream transcriptional activation [57]. Upon oxidative stress, this complex dissociates, resulting in the liberation and subsequent nuclear translocation of Nrf2. The antioxidant defense system was subsequently activated, leading to the transcription of a series of downstream antioxidant genes, like Heme oxygenase-1(HO-1), SOD, or GSH-Px [58]. In this study, HAPH-1 upregulated the protein expression of Nrf2 and downregulated the protein expression of Keap1 in testicular tissue of TP-treated mice. The SOD or GSH-Px activities were also dramatically elevated by HAPH-1 treatment, accompanied by enhanced testicular T-AOC and decreased MDA. These results indicated the activation of the Keap1-Nrf2 signaling pathway. Qin et al. [51] reported that melatonin alleviates TP-induced oxidative stress by activating Nrf2, thereby contributing to the reduction of TP-induced male reproductive damage. Zhang et al. [33] demonstrated that oyster peptides could upregulate the expression of Nrf2 in the testes, thereby activating the Keap1-Nrf2 signaling pathway and ameliorating TP-induced reproductive damage in mice. These observations are in agreement with our present results. Our results indicated that the antioxidant peptides from *H. abdominalis* might ameliorate testicular tissue oxidative injury and thereby further alleviate male reproductive damage by activating the Keap1-Nrf2 pathway.

Apoptosis represents a common process of programmed cellular death [59]. Previous evidence indicated that TP can induce oxidative stress in spermatocytes, activate apoptotic signaling, and ultimately result in apoptosis of testicular cells, thereby impairing spermatogenesis and normal physiological functions of sperm [60]. Cell apoptosis is modulated by anti-apoptotic proteins like BCL-2 and pro-apoptotic proteins such as BAX [51]. These two molecules constitute a key regulatory pair governing apoptotic progression. An elevated Bax/Bcl-2 ratio can trigger the activation of caspase-3 in its cleaved form, thereby facilitating the apoptotic process [61]. The present study demonstrated that HAPH-1 treatment markedly upregulated Bcl-2 expression and downregulated Bax expression, which in turn suppressed caspase-3 activation and the caspase cascade, thus reducing testicular cell apoptosis. Yan et al. [62] demonstrated that heat-transformed saponins (HTS) inhibit cellular apoptosis through the Bcl-2/Bax/Caspase-3 signaling pathway, thereby effectively ameliorating cyclophosphamide-induced male reproductive injury. These results are consistent with our study, indicating that HAPH-1 can inhibit cell apoptosis through the Bcl-2/Bax/Caspase-3 pathway and effectively alleviate TP-induced male reproductive damage. Furthermore, these findings are consistent with our observations from testicular histopathological sections and TUNEL apoptosis detection.

The rapid advancement of bioinformatics allows researchers to utilize online databases for the prediction of biological properties of peptides. The HAPH-1 fraction was characterized by LC-MS/MS, and a total of 4033 peptides were identified. Subsequently, a series of online bioinformatic tools, including PeptideRanker, ToxinPred, and other related platforms [63], were used to screen and identify 50 non-toxic, non-sensitizing peptides with antioxidant potential. Molecular docking analysis with Keap1 was performed to screen for highly active peptides [64], and three peptides with the highest binding affinities were identified: GPAPWGF, FPALLF, and LPLPLF. Molecular docking results demonstrated that GPAPWGF formed hydrogen bonds with the amino acid residues Asn414, Arg380, Asn387, Ser431, Arg415, and Ser363, as well as hydrophobic interactions with Ile461, Asn387, Phe478, Tyr334, Phe577, and Tyr572. FPALLF established hydrogen bonds with Gly433, Arg380, Asn387, Asp389, Ser431, and hydrophobic interactions with Ile461, Asn387, Phe478, Tyr334. LPLPLF formed hydrogen bonds with Arg415, Gly364, Arg380, together with hydrophobic interactions with Phe577, Tyr572, Tyr334, Arg415. The amino acid residues Arg415, Arg380, Ser508, and Ser363 are identified as critical binding sites for Keap1 [65]; all three peptides can bind to the key residues of Keap1. In addition, the binding energies of GPAPWGF, FPALLF, and LPLPLF with DPPH free radicals were −4.8 kcal/mol, −3.8 kcal/mol, and −3.3 kcal/mol, respectively; the corresponding binding energies with ABTS free radicals were −3.6 kcal/mol, −3.2 kcal/mol, and −3.4 kcal/mol, respectively. Finally, the three purified peptides were synthesized, and their IC_50_ values for DPPH, ABTS, and hydroxyl radical scavenging, as well as reducing power at 2 mg/mL, were determined. The IC_50_ values for free radical scavenging and the reducing power of GPAPWGF, FPALLF, and LPLPLF were determined. For DPPH radicals, the IC_50_ values were 1.48 ± 0.13, 2.32 ± 0.18, and 2.21 ± 0.18 mg/mL, respectively. For ABTS radicals, the IC_50_ values were 0.386 ± 0.01, 0.416 ± 0.03, and 0.561 ± 0.02 mg/mL, respectively. For hydroxyl radicals, the IC_50_ values were 1.259 ± 0.04, 0.857 ± 0.03, and 0.918 ± 0.03 mg/mL, respectively. The reducing power values were 0.675 ± 0.01, 0.709 ± 0.02, and 0.603 ± 0.03, respectively. The differences in IC_50_ values and reducing power among these peptides reflected their distinct antioxidant capacities. In vitro antioxidant activity of all three peptides was higher than that of HAPH-1. Most reported bioactive peptides exhibit IC_50_ values in the ranges of 1.30–2.40 mg/mL (DPPH), 0.75–0.98 mg/mL (ABTS), and 1.07–1.43 mg/mL (hydroxyl radical) for free radical scavenging [66]. Overall, the three target peptides in this work displayed remarkably superior antioxidant capacity, which endows them with higher application value in related fields.

## 5. Conclusions

In summary, our findings demonstrate that HHP-assisted enzymatic hydrolysis significantly improves the peptide yield and antioxidant activity of *H. abdominalis* hydrolysates. The optimal hydrolysis parameters were systematically optimized using single-factor experiments combined with response surface methodology. The HAPH-1 fraction, purified from *H. abdominalis* hydrolysates via ultrafiltration, exhibits excellent antioxidant activity and superior stability. Meanwhile, HAPH-1 effectively mitigates testicular injury, oxidative stress, and inflammation in TP-treated mice. Further mechanistic studies indicate that its protective effects are closely associated with the regulation of the Keap1-Nrf2 signaling pathway and the Bax/Bcl-2/Caspase-3 apoptotic pathway. In addition, three novel antioxidant peptides—GPAPWGF, FPALLF, and LPLPLF—were identified using computer-aided virtual screening and molecular docking. These peptides form strong interactions with Keap1 through hydrophobic forces and hydrogen bonds, contributing to their prominent antioxidant capacity. Overall, the present study successfully identified novel natural antioxidant peptides, laying a robust theoretical foundation for the high-value exploitation of *H. abdominalis*. Furthermore, this study offers a promising scientific basis for the development of innovative antioxidant functional foods and bioactive ingredients.

## Figures and Tables

**Table 1:** Analysis of the variance of the fitted second-order polynomial models.

	Peptide Yield/%						DPPH Radicals Scavenging Capacity/%					
	Sum of Squares	df	Mean Square	*F*-Value	*p*-Value	Significant	Sum of Squares	df	Mean Square	*F*-Value	*p*-Value	Significant
Model	528.00	9	58.67	152.65	<0.0001	**	146.82	9	16.31	242.23	<0.0001	**
A’	0.30	1	0.30	0.78	0.41		1.84	1	1.84	27.31	<0.0001	**
B’	0.74	1	0.74	1.93	0.21		0.17	1	0.17	2.54	0.16	
C’	5.09	1	5.09	13.24	0.01	**	1.96	1	1.96	29.03	<0.0001	**
A’B’	0.37	1	0.37	0.97	0.36		0.00	1	0.00	0.05	0.84	
A’C’	0.68	1	0.68	1.77	0.23		0.83	1	0.83	12.30	0.01	**
B’C’	2.20	1	2.20	5.73	0.05	*	0.69	1	0.69	10.22	0.02	**
A’^2^	173.51	1	173.51	451.49	<0.0001	**	51.35	1	51.35	762.42	<0.0001	**
B’^2^	149.59	1	149.59	389.25	<0.0001	**	30.13	1	30.13	447.32	<0.0001	**
C’^2^	141.05	1	141.05	367.01	<0.0001	**	45.21	1	45.21	671.33	<0.0001	**
Residual	2.69	7	0.38				0.47	7	0.07			
Lack of Fit	1.31	3	0.44	1.26	0.40	not significant	0.22	3	0.07	1.15	0.43	not significant
Pure Error	1.38	4	0.35				0.25	4	0.06			
Cor Total	530.69	16					147.29	16				
R^2^	0.9949						0.9968					
R^2^_Adj_	0.9884						0.9927					
C.V. %	0.85						0.47					

* indicates significant effect (*p* < 0.05), ** indicates highly significant effect (*p* < 0.01).

**Table 2:** Bioactivity prediction and molecular docking of peptides.

Number	Peptide	Binding Energy	Score	pI	AA%	GRAVY	Allergenicity	Toxicity
1	GPAPWGF	−11.1	0.99	5.52	100.00%	−0.043	Non-allergen	Non-toxin
2	FPALLF	−9.9	0.97	5.52	100.00%	2.233	Non-allergen	Non-toxin
3	FPAILF	−8.1	0.96	5.41	83.33%	2.35	Non-allergen	Non-toxin
4	LPLPLF	−9.5	0.94	5.46	100.00%	1.833	Non-allergen	Non-toxin
5	FQPSFL	−7.7	0.94	5.52	66.67%	0.583	Non-allergen	Non-toxin
6	PEFPFG	−8.8	0.94	4	62.50%	−0.25	Non-allergen	Non-toxin
7	RGFDPFMNL	−9.1	0.93	5.84	50.00%	−0.244	Non-allergen	Non-toxin
8	IPLPLF	−7.8	0.93	5.46	83.33%	1.95	Non-allergen	Non-toxin
9	PLPAFL	−7.7	0.93	5.63	100.00%	1.5	Non-allergen	Non-toxin
10	KPGLPFDF	−9	0.92	6.44	66.67%	−0.2	Non-allergen	Non-toxin
11	LPLPIF	−8.4	0.91	5.52	83.33%	1.95	Non-allergen	Non-toxin
12	ALMLLF	−6.6	0.91	5.57	66.67%	2.983	Non-allergen	Non-toxin
13	NHFYHF	−8.6	0.91	5.92	100.00%	−0.933	Non-allergen	Non-toxin
14	ALMILF	−7.6	0.90	5.57	100.00%	3.1	Non-allergen	Non-toxin
15	FNPVPMMKL	−8.2	0.89	8.75	50.00%	0.44	Non-allergen	Non-toxin
16	WDLDHGF	−8.4	0.89	4.2	77.78%	−0.7	Non-allergen	Non-toxin
17	TVFQPFF	−8.8	0.89	5.79	75.00%	0.971	Non-allergen	Non-toxin
18	ISFGNF	−8.2	0.89	5.52	85.71%	0.9	Non-allergen	Non-toxin
19	GIDYLF	−8.5	0.89	3.8	100.00%	0.983	Non-allergen	Non-toxin
20	SFNQIRF	−7.9	0.88	9.47	100.00%	−0.314	Non-allergen	Non-toxin
21	YKLPPGFL	−8.5	0.88	8.59	33.33%	0.2	Non-allergen	Non-toxin
22	WEQWAKF	−9.2	0.88	6	85.71%	−1.157	Non-allergen	Non-toxin
23	LSFGNF	−8.9	0.88	5.52	71.43%	0.783	Non-allergen	Non-toxin
24	FQPSFI	−9	0.88	5.52	75.00%	0.7	Non-allergen	Non-toxin
25	EEGMPFF	−8.1	0.87	3.8	66.67%	−0.214	Non-allergen	Non-toxin
26	HGGGFDIGF	−9.2	0.87	5.08	71.43%	0.2	Non-allergen	Non-toxin
27	QVFPMNPPKF	−8.3	0.87	8.75	100.00%	−0.4	Non-allergen	Non-toxin
28	FGAFGNL	−8.8	0.87	5.52	77.78%	0.986	Non-allergen	Non-toxin
29	LFQPSF	−7.9	0.85	5.52	57.14%	0.583	Non-allergen	Non-toxin
30	GYALPHAIMRL	−7.9	0.85	8.75	66.67%	0.6	Non-allergen	Non-toxin
31	APQLMIPIKF	−7.6	0.85	8.8	71.43%	0.87	Non-allergen	Non-toxin
32	PLPAFI	−9.1	0.85	5.96	66.67%	1.617	Non-allergen	Non-toxin
33	FSSQAYAPAKNLLF	−7	0.84	8.59	66.67%	0.229	Non-allergen	Non-toxin
34	PAPAAARIPKKLLL	−7.9	0.84	11.17	85.71%	0.429	Non-allergen	Non-toxin
35	GLSVLPLPRL	−8.1	0.84	9.75	42.86%	1.05	Non-allergen	Non-toxin
36	HINPRF	−8.4	0.83	9.76	75.00%	−0.917	Non-allergen	Non-toxin
37	SAPFQKPKPAKGKAEAHF	−7.4	0.83	10	85.71%	−1.056	Non-allergen	Non-toxin
38	VPISGWHGDNML	−8.3	0.83	5.08	57.14%	0.008	Non-allergen	Non-toxin
39	AYAPAKNLLF	−8.5	0.83	8.63	66.67%	0.55	Non-allergen	Non-toxin
40	ALRPPLDF	−8.5	0.83	5.88	66.67%	0.125	Non-allergen	Non-toxin
41	ERFLPML	−7.6	0.83	6.1	50.00%	0.386	Non-allergen	Non-toxin
42	FLLGGL	−7.1	0.83	5.52	71.43%	2.233	Non-allergen	Non-toxin
43	DIMGKPPRSPKEPLISGWL	−6.3	0.82	8.59	77.78%	−0.553	Non-allergen	Non-toxin
44	RGAPMGM	−8.3	0.82	9.75	66.67%	−0.186	Non-allergen	Non-toxin
45	FAHLLPRHL	−8.7	0.81	9.76	70.00%	0.389	Non-allergen	Non-toxin
46	LGLGDMF	−8.7	0.81	3.8	85.71%	1.143	Non-allergen	Non-toxin
47	LFLGGL	−8	0.80	5.52	57.14%	2.233	Non-allergen	Non-toxin
48	KSAPFQKPKPAKGKAEAHF	−6.8	0.80	10.18	57.14%	−1.205	Non-allergen	Non-toxin
49	LPGLYLL	−8	0.80	5.52	63.64%	1.7	Non-allergen	Non-toxin
50	ALFQPSF	−8.7	0.80	5.57	66.67%	0.757	Non-allergen	Non-toxin

**Table 3:** Antioxidant activity of P1, P2, and P4.

Group	IC_50_ (mg/mL)			The Reducing Power
	DPPH Radicals	ABTS Radicals	Hydroxyl Radicals	(2 mg/mL)
HAPH-1	3.043 ± 0.05 ^a^	0.763 ± 0.06 ^a^	1.259 ± 0.04 ^a^	0.332 ± 0.01 ^d^
P1	1.476 ± 0.13 ^c^	0.386 ± 0.01 ^c^	0.686 ± 0.03 ^c^	0.675 ± 0.01 ^b^
P2	2.316 ± 0.18 ^b^	0.416 ± 0.03 ^c^	0.857 ± 0.03 ^b^	0.709 ± 0.02 ^b^
P4	2.212 ± 0.18 ^b^	0.561 ± 0.02 ^b^	0.918 ± 0.03 ^b^	0.603 ± 0.03 ^c^
GSH	1.003 ± 0.03 ^d^	0.074 ± 0.01 ^d^	0.195 ± 0.02 ^d^	1.721 ± 0.02 ^a^

Data are presented as mean ± SD (n = 3). Different lowercase letters (a–d) within the same column indicate significant differences at *p* < 0.05.

## Data Availability

The datasets generated and/or analyzed during the current study are available from the corresponding author upon reasonable request.

## Supplementary Materials

The following supporting information can be downloaded at: https://www.mdpi.com/article/10.3390/antiox15101338/s1; Supplementary Data S1:All identified peptide sequences.; Table S1: Plackett–Burman factorial design for determination of significant factors; Table S2: Effect analysis of response surface methodology experiments; Table S3: Interaction forces between peptides and Keap1; Table S4: The docking binding energies of P1, P2, and P4.
