# Supplementary material for: Preparation of Antioxidant Peptides from Hippocampus Abdominalis via High Hydrostatic Pressure-Assisted Enzymolysis: Evaluation of Their Protective Effects Against Male Reproductive Dysfunction in Mice
Source: Antioxidants (Basel). 2026 Oct 16;15(10):1338. doi: 10.3390/antiox15101338 (PMC13701427; doi:10.3390/antiox15101338)
Supplement: Supplementary file 1 [file antioxidants-15-01338-s001.zip › Supplementary Tables.pdf]

Table S1 Plackett–Burman factorial design for determination of significant factors

Source	Peptide yield	DPPH radicals scavenging capacity
Model	0.0387*	0.0016**
A-Enzyme to substrate ratio	0.015*	0.0001**
B-Enzymatic hydrolysis time	0.0632	0.823
C-solid liquid rate	0.0325*	0.0304*
D-HHP pressure	0.7093	0.5707
E-HHP time	0.004**	0.243

Table S2 Effect analysis of response surface methodology experiments

Number	A: Enzyme to substrate ratio (%)	B: Solid liquid rate (g/mL)	C:HHP time (min)	Y_1: Peptide yield/%	Y_2: DPPH radicals scavenging capacity/%
1	2	20	8	69.17	54.50
2	4	20	8	69.58	53.59
3	2	30	8	69.84	54.42
4	4	30	8	69.03	53.62
5	2	25	6	70.04	54.01
6	4	25	6	70.29	53.85
7	2	25	10	69.69	53.91
8	4	25	10	68.29	51.94
9	3	20	6	72.42	54.85
10	3	30	6	69.66	54.62
11	3	20	10	68.93	53.04
12	3	30	10	69.13	54.47
13	3	25	8	82.22	60.26
14	3	25	8	82.33	60.02
15	3	25	8	82.01	59.88

16	3	25	8	81.43	60.52
17	3	25	8	80.93	60.31

Table S3 Interaction forces between peptides and Keap1

Peptide	Hydrogen Bonds		Hydrophobic Interaction	
	Quantity	Binding Sites	Quantity	Binding Sites
GPAPWGF	10	Asn414, Arg380, Asn387, Ser431, Arg415 and Ser363	9	Ile461, Asn387, Phe478, Tyr334, Phe577, Tyr572
FPALLF	11	Gly433, Arg380, Asn387, Asp389, Ser431	6	Ile461, Asn387, Phe478, Tyr334
LPLPLF	5	Arg415, Gly364, Arg380	6	Phe577, Tyr572, Tyr334, Arg415

Table S4 The docking binding energies of P1, P2, and P4

Peptide	Binding Energy（kcal/mol）		
	Keap1	DPPH radicals	ABTS radicals
P1	-11.1	-4.8	-3.6
P2	-9.9	-3.8	-3.2
P4	-9.5	-3.3	-3.4
